# Evaluation of Pulsed Electric Field and Conventional Thermal Processing for Microbial Inactivation in Thai Orange Juice

**DOI:** 10.3390/foods11081102

**Published:** 2022-04-12

**Authors:** Chatchawan Kantala, Supakiat Supasin, Panich Intra, Phadungsak Rattanadecho

**Affiliations:** 1 Department of Mechanical Engineering, Faculty of Engineering, Thammasat University, Rangsit Centre, Pathum Thani 12121, Thailand; chatchawan_kantala@yahoo.com (C.K.); supakiat_31@hotmail.com (S.S.); 2 Research Unit of Applied Electric Field in Engineering (RUEE), College of Integrated Science and Technology, Rajamangala University of Technology Lanna, Chiang Mai 50220, Thailand; panich_intra@yahoo.com

**Keywords:** Thai orange juice, pulsed electric field, microbial inactivation, conventional thermal pasteurization

## Abstract

A pulsed electric field (PEF) is a technology used for microbial inactivation in food and beverages. This study aimed to examine the effect of PEF treatment on microbial inactivation and quality parameters in Thai orange juice (TOJ). The results showed that PEF and conventional thermal pasteurization (CTP) can be performed for inactivation of *Staphylococcus aureus* and *Escherichia coli* in TOJ. A 5-log reduction was obtained after 10 pulses of PEF treatment when using and electrical field strength of 30 kV cm^−1^, and the microbial inactivation by the PEF treatment resulted from the electroporation more than the temperature. Moreover, PEF treatment affects the quality parameters less than CTP. Moreover, PEF treatment did not affect the TOJ quality parameters such as pH, commission international de l’eclairage (CIE), viscosity, and total soluble solid (TSS), but saved vitamin C and all sugar and all mineral (sucrose, glucose, fructose, sodium, lithium, potassium, magnesium, and calcium) values more than CTP treatment.

## 1. Introduction

At present, the preservation of liquid or fruit juices uses a pasteurization process to inactivate the bacteria so as to prolong the shelf life as long as possible. In the pasteurization process, heat is used to inactivate microorganisms, ranging from 70–100 °C for 15 to 30 min. The disadvantage of using heat to inactivate microorganisms is that vitamins or substances that are sensitive to heat are lost, along with taste, odour, and colour. Therefore, after the heat treatment, it is necessary to add vitamins and various nutrients to provide benefits to consumers, but this also results in increased costs. So, the process of inhibiting microorganisms without using heat, such as through high-pressure cold sterilization or a pulsed electric field, is another option for heat-sensitive liquid foods or juices with vitamins or nutritional value, also being able to preserve the flavour, smell, and colour, as nowadays, consumers are drinking more fruit juices such as orange juice, coconut water, apple juice, grape juice, fruit mixtures, and others [1,2,3,4].

Pulsed electric field (PEF) technology, with short duration, high-voltage pulses and a high-intensity electric field, can be applied to food and beverages at temperatures below that of conventional thermal processing (CTP), and it decreases the contaminant microorganisms without thoroughly affecting the food’s quality [5,6,7]. A PEF processing system is composed of an energy storage capacitor bank, high-voltage power source, treatment chamber, pulse controller, charging resistor and discharge switch [8,9,10,11]. In food processing, PEF is used for many applications such as extracting bioactive compounds from herbs [12,13], food preservation, and microbial inactivation. Microbial inactivation by PEF is applied in juice beverages comprising apple juice, mixed fruit juice, and orange juice, etc. Previous studies reported that PEF has potentially use for microbial inactivation in juice. Timmerman et al. [14] compared the differences of the microbial inactivation methods comprising mild heat pasteurization, high pressure processing, and PEF. The result showed that the application of PEF with an electric field strength of 23 kV cm^−1^ at a 90 Hz frequency could be applied for the inhibition of various microbials such as *E. coli*, Enterobacteriaceae, yeast, and mould. Mosqueda-Melgar et al. [15] applied high-intensity PEF for microorganism inactivation in several juices, namely, apple, pear, tomato, strawberry, and orange. They found that the number of mesophilic and psychrophilic bacteria and mould and yeast will be reduced after treated with an electric field strength of 80 kV cm^−1^ and a treatment time of 60 µs with and without an antibacterial agent. Therefore, PEF is an interesting technique for microbial inactivation in orange juice that is problematic in the Thai juice industry. However, the quality of juice should not be changed. Sanchez-Moreno et al. [16] studied the effect of PEF on the quality of orange juice. They applied PEF with 35 kV cm^−1^ of electric field strength and 750 µs of treatment time into orange juice and compared the physicochemical characteristics of orange juice with other techniques. The result illustrated that PEF had high efficiency for preserving bioactive compound in orange juice. Cortes et al. [1] also documented that the effect of PEF on the colour of orange juice. The result confirmed that PEF with electric field 30 kV cm^−1^ and treatment time 100 µs has colour differences of orange juice lower than the pasteurized juice. Consequently, this study aimed to operate a PEF system and investigate the PEF parameters electric field strength, low treatment time, and pulse number for the treatment of Thai orange juice (TOJ) to inactivate microbials. Microbial inactivation and TOJ quality were investigated and compared between the CTP and PEF techniques for food processing applications.

Thailand (2018) had a total fruit juice export value of USD 572 million to the United States, Europe, Japan, China, and ASEAN. The juice of *Citrus reticulata* oranges, in Thai, called “Sai Nam Peung”, is dominant and more favoured than the other juices manufactured in Thailand. Many researchers have applied the PEF technique for microbial inactivation in oranges of different sources and species [17,18,19]. However, this research focused on *C. reticulata* oranges cultivated in Northern Thailand and the main problem of spoiled orange juice created in the manufacturing process.

## 2. Materials and Methods

### 2.1. Materials

TOJ purchased from a local supermarket has conductivity of 3.56 mS cm^−1^ at ambient temperature. The TOJ was stored at 4 °C. *Staphylococcus aureus* TISTR 2329 and *Escherichia coli* TISTR 117 were ordered from the Thailand Institute of Scientific and Technological Research (TISTR), Thailand. All operations were conducted in a room at an ambient temperature of 25 ± 2 °C.

### 2.2. PEF Operation

**Figure 1:**
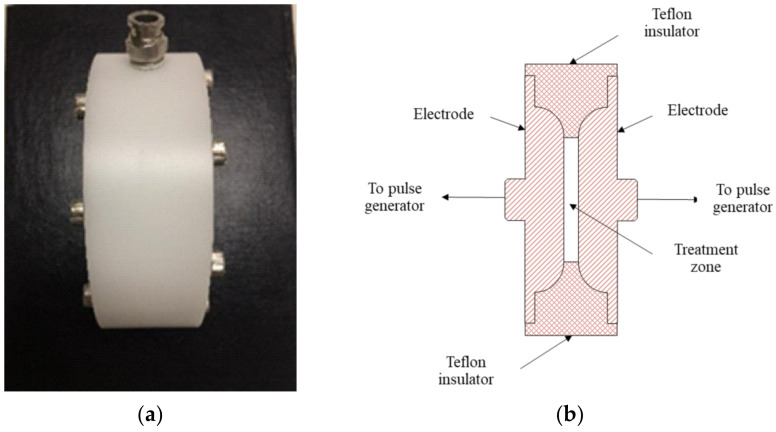
Treatment chamber for the study: (**a**) photograph of treatment chamber, (**b**) schematic diagram of chamber.

The PEF design and analysis followed that of Kantala et al. [20]; the system includes a rectifier circuit, alternating current (AC) power input, energy storage capacitor, direct current (DC) high-voltage power, and pulse frequency control. A digital oscilloscope (TDS 210, Tektronix, Sausalito, CA, USA) was applied to determine the pulse waveform. The input and output of voltage were determined using a digital multimetre (289 True-rms, Fluke, Everett, WA, USA) and a high-voltage probe (80K-40, Fluke, Everett, WA, USA). After the sample was treated with PEF, its temperature was estimated using a thermometer (Fluke FoodPro Plus Food Safety Thermometer, Everett, WA, USA). We also operated the treatment chamber, composed of two substantially parallel stainless-steel electrodes (316 L grade) with a gap of 5 mm and an insulator (Teflon). The chamber volume was about 80 cm^3^, and the area (treatment zone) was 36 cm^2^ (Figure 1).

### 2.3. PEF Treatment

**Figure 2:**
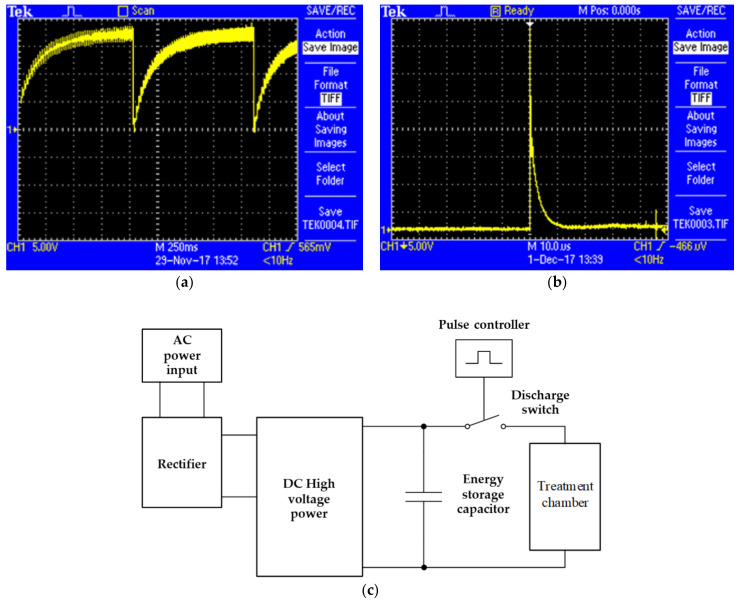
Pulse waveforms: (**a**) charging, (**b**) discharging of voltages, (**c**) PEF operation.

PEF was used for microbial inactivation in TOJ. The concentration of microorganism in orange juice before the PEF Treatment (initial concentration) was 8.4 × 10^5^ CFU mL^−1^ for *S. aureus* and 8.9 × 10^5^ CFU mL^−1^ for *E. coli*, and a volume of 100 mL of inoculated orange juice was prepared for each experiment. After that, TOJ was treated with PEF at different conditions. The electric field strength was set at 20, 30, and 40 kV cm^−1^, and the pulse numbers were 0, 10, 20, 30, 40, and 50, respectively. Treated TOJ was kept in a refrigerator at 4 °C. The PEF treatment parameters were composted of frequency, pulse width, pulse number or treatment time, and pulse waveforms (Table 1, Figure 2).

### 2.4. CTP Treatment

**Figure 3:**
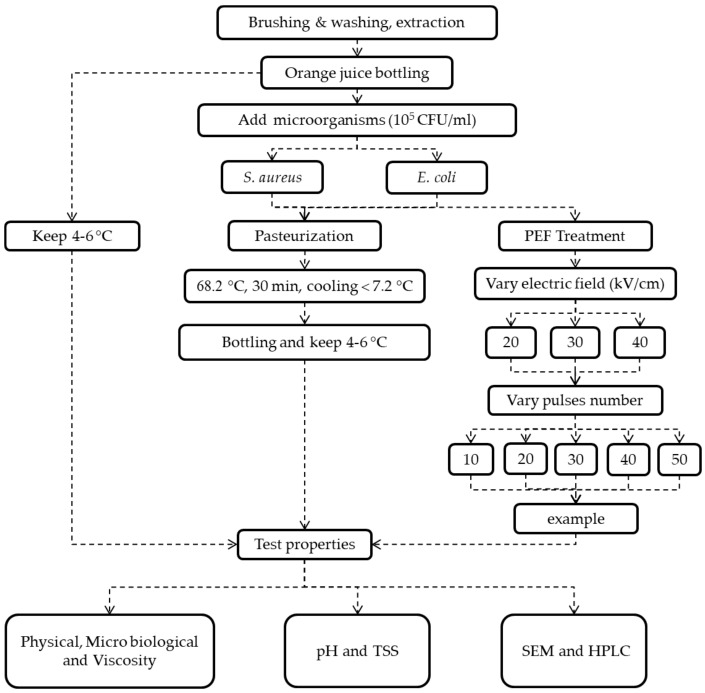
Flow diagram of PEF for microbial inactivation.

Spherical and rod-shaped microbes, respectively, *S. aureus* [21] (8.4 × 10^5^ CFU mL^−1^) and *E. coli* (8.9 × 10^5^ CFU mL^−1^), were added into 100 mL of fresh TOJ. The mixed TOJ was treated by thermal processing as described by Solomon et al. [22] at 68.2 °C for 30 min, then thoroughly cooled down to 7.2 °C (Figure 3); treated TOJ was kept in a refrigerator at 4 °C.

### 2.5. Sample Analysis

#### 2.5.1. Microbiology

After PEF and CTP treatment, treated-TOJ was analysed its properties comprising microbiology and qualities. In microbiology, the number of microbial by the spread plate technique. Sample (0.1 mL) was put on nutrient agar and incubated at 37.5 °C for 24 h. The viable colonies in treated-TOJ was counted and compared with untreated-TOJ.

#### 2.5.2. Scanning Electron Microscopy (SEM)

The cell morphology of the microbes in untreated and treated TOJ was examined and compared by SEM (JSM-5910LV, JEOL, Tokyo, Japan). Samples were prepared by chemical tissue stabilization techniques using glutaraldehyde. The samples were dried with a critical point dryer (CPD; K850, Quorum, Mytchett, UK). For SEM analysis, a few milligrams of sample was attached to carbon tape on a brass stub and sputtered with gold for 45 s. The SEM was operated at an accelerating voltage of 15 kV and images were taken.

#### 2.5.3. TOJ Quality

The quality of untreated and treated TOJ was determined using a thermometer (51-2, Fluke, Everett, WA, USA), pH meter (LAQUA twin pH 33, HORIBA, Kyoto, Japan), total soluble solids (TSS) hand refractometer (MASTER-20M, ATAGO, Saitama, Japan) and viscometer (LVDV-E, Brookfield, Brisbane, Queensland, Australia). The colour of untreated and treated TOJ was determined by measuring the CIE values composed of brightness (L*), redness (a*) and yellowness (b*) using a colour meter (A60-1011-610, ColorQuest XE, Reston, VA, USA).

#### 2.5.4. TOJ Composition

The chemical composition of untreated and treated TOJ was compared by separation and quantification of sugars, vitamin C and minerals using high-performance liquid chromatography (HPLC; SPD-20A UV-Vis detector, Shimadzu, CA, USA). For vitamin C analysis, a 5 µm C18 column (Shimpack GIST; 6 × 150 mm) was used with a mobile phase composed of 25 mM potassium dihydrogen phosphate (KH_2_PO_4_) in water and 1 mL of phosphoric acid (H_3_PO_4_) at a flow rate of 1.0 mL min^−1^. The column oven temperature was 40 °C. A photodiode array (PDA) detector was used at a wavelength of 243 nm and injection volume of 5 µL. Sugars, comprising sucrose, glucose and fructose, were analysed using a 7.8 × 300 mm column (Aminex HPX-87N) with a mobile phase of water at a flow rate of 0.6 mL min^−1^ and column oven temperature of 60 °C. A reflective index detector was used and the injection volume was 20 µL. The mineral composition, including lithium, sodium, potassium, magnesium and calcium, was analysed using a 4 × 250 mm column (Ionpac CS12A) with a mobile phase of 3.5 mM sulphuric acid (H_2_SO_4_) at a flow rate of 1.0 mL min^−1^ and column oven temperature of 40 °C. A conductivity detector was used and the injection volume was 50 µL.

### 2.6. Statistical Analysis

Data are presented as averages ± standard deviations. A multivariate general linear model (GLM) and one-way analysis of variance (ANOVA) were utilized to compare the treatments. Tukey’s HSD was applied for multiple comparisons of each treatment. The data analysis was done using SPSS program version 24.0.

## 3. Results and Discussion

### 3.1. Microbial Inactivation by PEF and CTP Treatments

**Figure 4:**
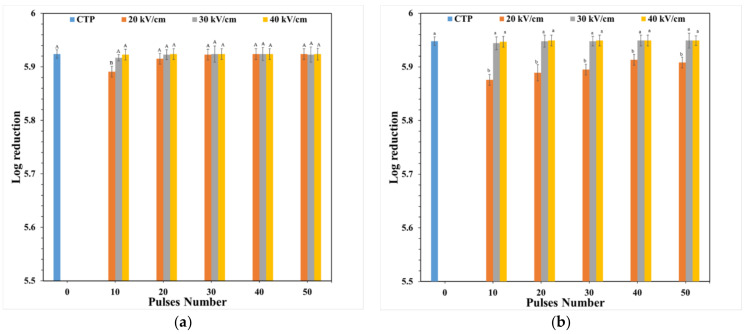
Log reduction of *S. aureus* (**a**) and *E. coli* (**b**). Values are presented as the means ± standard deviations (SD) (*n* = 3); the different capital letters (A and B) and lowercase letters (a and b) on each bar indicated significant differences (one-way ANOVA and Turkey’s HSD, *p* ≤ 0.05) among samples.

Figure 4 shows that PEF and CTP treatments affected the number of microbial cells. The CTP technique decreased the number of viable of *S. aureus* and *E. coli* cells by 5.891 and 5.949 log, respectively. This demonstrates that CTP at 68.2 °C for 30 min can be used for microbial inactivation (5.936 log). Thai-designed PEF can be used for microbial inactivation: PEF treatment at 20 kV cm^−1^ and 10–50 pulses decreased the number of *S. aureus* cells by 5.891–5.924 log; at 30 kV cm^−1^ and 10–50 pulses by 5.917–5.923 log; and at 40 kV cm^−1^ and 10–50 pulses by 5.923–5.924 log. The number of *E. coli* cells was decreased by 5.876–5.908 log after PEF treatment at 20 kV cm^−1^ and 10–50 pulses; 5.944–5.949 log at 30 kV cm^−1^ and 10–50 pulses; and 5.947–5.949 log at 40 kV cm^−1^ and 10–50 pulses. These results are similar to those of the study of Mosqueda-Melgar et al. [15] who found that increasing electric field strength and treatment time or pulse number increases microbial inactivation. This study demonstrated that it is difficult to inactivate *E. coli* in orange juice as it is rod-shaped, a cell morphology which requires an electric field strength approximately 15% stronger than for elliptical or circular cells [23]. Moreover, the electric field strength and pulse number (30 kV cm^−1^, 10 pulsed for inactivation of *S. aureus* and *E. coli*) showed the high efficiency of microbial inactivation, similar to that observed in the study of Gupta et al. [24]. Microbial inactivation by PEF results from electroporation, while CTP involves thermal cell membrane destruction [25]. These results are similar to those of the study of Timmermans et al. [14] who showed that PEF and CTP similarly affect microbial inactivation in orange juice. Therefore, our study exhibited that this PEF system has similar potential for microbial inactivation in orange juice as the CTP technique.

### 3.2. Microbial Cell Morphology

**Figure 5:**
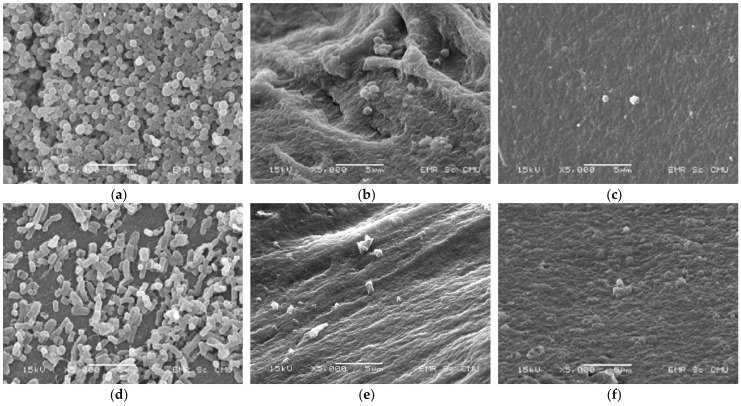
SEM micrographs of untreated *S. aureus* (**a**), CTP-treated *S. aureus* (**b**), *S. aureus* treated with PEF at 40 kV cm^−1^ and 40 pulses (**c**), untreated *E. coli* (**d**), CTP-treated *E. coli* (**e**) and *E. coli* treated with PEF at 40 kV cm^−1^ and 40 pulses (**f**).

SEM micrographs were used to confirm the inactivation of *S. aureus* and *E. coli* before (control) and after treatment with PEF and CTP. The results show that PEF and CTP affected the microbial count (Figure 5), but did not change the morphology of *S. aureus* (Figure 5b,c). These results suggest that CTP and PEF can be used for microbial inactivation. However, the number of microbes was required by the viable cell count method. Furthermore, the cell ultrastructure comprising inter- and intra-cellular structure organization should be described by transmission electron microscope (TEM) in further study as stated by Chittapun et al. [26].

### 3.3. TOJ Quality

Another key variable in PEF treatment is temperature. The results show that the increment of electric field strength and pulse number raised the temperature. TOJ treated with PEF showed the highest temperature of 43.6 ± 0.52 °C when using 40 kV cm^−1^ and 50 pulses and the lowest temperature of 26.1 ± 0.48 °C for 20 kV cm^−1^ and 10 pulses, temperature increase, as a consequence of Joule heating, such as specific energy input per pulse, mass of sample, electrical conductivity of sample, electric field strength, treatment times and electrode area [27]. After treating TOJ with CTP, the temperature was increased by thermal conduction to 68.2 ± 0.51 °C (Table 2). Therefore, the CTP technique generated a higher temperature than the PEF technique. The high temperature resulted from the transfer of heat energy [28]; thermal processing involves the transfer of heat energy my mechanisms such as conduction, convection and radiation. However, PEF also causes a high temperature for microbial inactivation [29], PEF having potential as a rapid heating technology based on ohmic heating for microbial inactivation. This study confirmed that PEF can increase the temperature of liquids such as orange juice. The results indicate that PEF has more potential than CTP as discussed above.

The energy (*Q*) can be calculated by the following Equation (1) [26], and depends on the capacitance of the storage capacitor, *C*, the initial charge voltage, *Vc*, the number of pulses, *n*, and the volume of the treatment chamber, *v*:

(1) $$Q=\frac{1}{2v}CV_{c}^{2}n$$

There were variations in the energy required for CTP and PEF treatments, showing that the energy required for PEF is less than for CTP treatment. The increment of PEF energy was affected by the electric field strength and pulse number. The maximum electric field strength of 40 kV cm^−1^ and maximum number of pulses (50) required 66.7 kJ L^−1^ for operation. The energy required for the CTP experiment was 160 kJ L^−1^.

**Figure 6:**
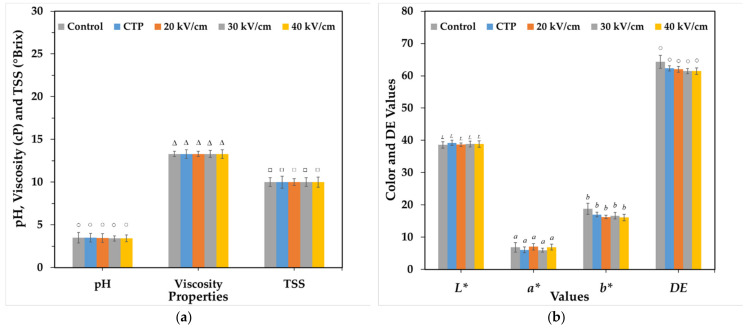
Variations in properties compared to control, after CTP and different PEF treatments: pH, viscosity and TSS (**a**) and L* a* b* colour coordinates and DE (**b**).

Moreover, the values of pH, TSS, viscosity and DE values are the sum of L* a* and b* values, which is more associated to consumer perception than singular L* a* or b* values in TOJ were not significantly different after applying CTP and PEF treatments (*p* < 0.05) (Figure 6). These results are similar to those of Timmermans et al. [14] who reported that qualitative measurements of °Brix, pH and dry matter content give stable results for mild heat pasteurization, high pressure and PEF. It has also been documented that there is no significant difference in pH between untreated and PEF-treated samples [30]. Chittapun et al. [26] reported that the application of PEF to distilled water can increase the pH, temperature and conductivity. Therefore, PEF and CTP treatments can affect pH, TSS, viscosity and DE values.

The quality of TOJ was estimated after PEF and CTP treatments by HPLC. The results showed that CTP and PEF treatments affected vitamin and nutrient values in TOJ. All ingredients in TOJ were not significantly different after PEF treatment at 20–40 kV cm^−1^ (*p* < 0.05), because the PEF technology as a non-thermal cell membrane permeabilization treatment [27]. CTP caused more reduction in all ingredients compared to PEF treatment (Table 3). This reduction resulted from the thermal effect. Temperature can affect the vitamin C content and aroma of oranges [16,25]. In this study, we focused on the vitamin C content that is the highest valuable compound in orange juice. These results indicated that PEF illustrated lower effect on vitamin C content than CTP technique. Furthermore, Sánchez-Moreno et al. [16] also reported that pasteurization showed higher vitamin C reduction than PEF technique. Therefore, a mild temperature should be used for orange juice treatment.

This study showed that PEF has potential for microbial inactivation and does not affect food quality. Moreover, the mild conditions for microbial inactivation in TOJ are an electric field strength of 30 kV cm^−1^ and pulse number of 10.

## 4. Conclusions

This study showed the design and operation of a PEF system for food processing. Both PEF and CTP techniques can be applied for the inactivation of *S. aureus* and *E. coli*. The results confirmed that this PEF system has potential similar to those of previous studies. The exceptional PEF conditions for microbial inactivation were an electric field strength of 30 kV cm^−1^ for 10 pulses that demonstrated a 5.9 log reduction in microorganism instead of microbial growth. The PEF technique demonstrated better performance than CTP in terms of the temperature and energy required for operation. No important differences regarding the quality were found in the values of the CIE, DE, pH, viscosity, TSS (°Brix). The quality of all sugars, all minerals and vitamin C between the untreated TOJ (control) and the TOJ treated by PEF and CTP treatment showed a small decrease, but a noticeable decrease compared with CTP. Therefore, this study suggested that the optimum condition for microbial inactivation in TOJ should use the PEF with an electric field strength of 30 kV cm^−1^ for 10 pulses. Moreover, this PEF condition was suitable for quality preservation in TOJ, especially vitamin C. This finding can be applied using a PEF system at industrial scale. Moreover, a cooling system should be applied in further study.

## Figures and Tables

**Table 1:** Parameters of PEF.

Parameters	Conditions
Electric field strength	20–40 kV cm^−1^
Pulse number	10–50 pulse
Pulse width	10 µs
Pulse wave form	Exponential decay
Frequency	1 Hz
Treatment time	100–500 µs

**Table 2:** Temperature of TOJ before and after treatment.

Pulse Number	Temperature (°C)			
	CTP Treatment	PEF Treatment (kV cm^−1^)		
		20	30	40
0 (control)	ambient temperature (25.0 ± 2.00)			
10		26.1 ± 0.48 ^a^	28.9 ± 0.22 ^a^	35.6 ± 0.30 ^a^
20		27.0 ± 0.45 ^b^	30.2 ± 0.62 ^b^	37.5 ± 0.35 ^b^
30	68.2 ± 0.51 ^f^	27.8 ± 0.53 ^c^	31.7 ± 0.45 ^c^	39.1 ± 0.41 ^c^
40		28.6 ± 0.51 ^d^	34.0 ± 0.37 ^d^	41.6 ± 0.62 ^d^
50		29.7 ± 0.50 ^e^	36.9 ± 0.75 ^e^	43.6 ± 0.52 ^e^

Mean values ± standard deviation (SD); *n* = 3. Mean values within a column with different superscript letters (a–f) are significantly different to the control (*p* < 0.05).

**Table 3:** Quality of TOJ ingredients before and after treatment.

Ingredients (ppm)	Control	CTP	PEF (kV cm^−1^) at 40 Pulses		
			20	30	40
Vitamin C (×10^2^)	3.8 ± 0.34 ^d^	3.1 ± 0.13 ^a^	3.5 ± 0.36 ^b^	3.5 ± 0.33 ^b^	3.4 ± 0.35 ^b^
Sucrose (×10^3^)	48.6 ± 2.47 ^d^	37.4 ± 1.58 ^a^	43.1 ± 2.15 ^c^	42.1 ± 2.52 ^b,c^	41.8 ± 2.78 ^b^
Glucose (×10^3^)	34.9 ± 1.26 ^d^	28.3 ± 2.20 ^a^	31.9 ± 1.97 ^b^	32.1 ± 2.50 ^c^	32.0 ± 1.91 ^c^
Fructose (×10^3^)	38.2 ± 2.04 ^c^	30.5 ± 1.05 ^a^	34.4 ± 2.78 ^b^	34.3 ± 1.69 ^b^	34.1 ± 2.45 ^b^
Lithium	23.4 ± 1.17 ^d^	19.2 ± 1.58 ^a^	21.8 ± 1.10 ^c^	22.2 ± 1.20 ^b,c^	20.9 ± 0.98 ^b^
Sodium	48.6 ± 1.65 ^d^	41.2 ± 1.42 ^a^	45.7 ± 2.04 ^c^	45.4 ± 1.12 ^c^	44.7 ± 2.09 ^b^
Potassium (×10^2^)	14.7 ± 1.19 ^c^	11.9 ± 1.95 ^a^	13.5 ± 1.47 ^b^	13.7 ± 1.83 ^b^	13.1 ± 1.32 ^b^
Magnesium	60.7 ± 1.26 ^c^	51.3 ± 2.11 ^a^	60.0 ± 1.01 ^c^	59.6 ± 2.63 ^c^	56.3 ± 1.81 ^b^
Calcium	81.6 ± 2.25 ^b^	80.0 ± 1.67 ^a^	81.5 ± 2.08 ^b^	81.6 ± 1.14 ^b^	81.3 ± 1.89 ^b^

Mean values ± standard deviation (SD); *n* = 3. Mean values within a row with different superscript letters (a–d) are significantly different to the control (*p* < 0.05).

## Data Availability

The data used to support the findings of study are included within the article.
